# Supplementary material for: The Hsp70 homolog Ssb affects ribosome biogenesis via the TORC1-Sch9 signaling pathway
Source: Nat Commun. 2017 Oct 16;8:937. doi: 10.1038/s41467-017-00635-z (PMC5643326; doi:10.1038/s41467-017-00635-z)
Supplement: Supplementary file 1 — Supplementary Information [file 41467_2017_635_MOESM1_ESM.pdf]

File Name: Supplementary Information
Description: Supplementary Figures, Supplementary Tables and Supplementary Notes

File Name: Peer Review File

Supplementary Notes

Supplementary Note 1. Sch9-5A is phosphorylated in an SNF1-dependent manner.

Upon glucose depletion, the Sch9-5A mutant (Fig. 1a) was shifted to more slowly migrating species termed Sch9-5A-S2* (S2*). Phosphorylation to S2* was dependent on SNF1, because it was not observed when Sch9-5A was expressed in a Δ*snf1* background (Supplementary Fig. 1a). Direct comparison of the Sch9-S2 and Sch9-5A-S2* species on phos-tag gels revealed that S2* migrated faster than S2 (Supplementary Fig. 1a). Because Sch9-5A lacks TORC1-dependent phosphorylation, this indicated that Sch9-S2 species had retained one or more of their TORC1-dependent phosphorylation sites after 10 minutes of glucose depletion (Supplementary Fig. 1c).

Supplementary Note 2. The Maf1 phosphorylation defect in Δ*ssb1*Δ*ssb2* cells does not depend on SNF1.

Since SNF1 is partly activated in glucose-grown Δ*ssb1*Δ*ssb2* cells [1,2], we tested if the Maf1 phosphorylation defect in glucose-grown Δ*ssb1*Δ*ssb2* cells was due to SNF1-mediated inhibition of TORC1 [3]. This was not the case, because Maf1 phosphorylation was not restored in a Δ*ssb1*Δ*ssb2*Δ*snf1* strain (Supplementary Fig. 3c and 3d). However, consistent with the observation that SNF1 was required for the dephosphorylation of the Sch9-CT upon glucose depletion (Fig. 1), Maf1 remained hyper-phosphorylated in Δ*snf1* cells upon glucose depletion (Supplementary Fig. 3c, 3d, and see also Discussion).

Supplementary Note 3. Validation of the Hsf1 antibody (α-Hsf1) for phos-tag analysis.

Because yeast Hsf1 is essential, a strain in which endogenous Hsf1 was replaced with Hsf1-GFP was employed to test α-Hsf1 specificity. α-Hsf1 efficiently detected both Hsf1 and Hsf1-GFP upon analysis via SDS-PAGE followed by immunoblotting (Supplementary Fig. 4a, left panel), however, Hsf1-GFP was only poorly detected upon analysis via phos-tag gels followed by immunoblotting (Supplementary Fig. 4a, right panel). This observation allowed us to employ Hsf1-GFP extracts as negative controls during phos-tag analysis. In phos-tag gels Hsf1 from glucose-grown cells formed two major species termed H1 and H2 (Supplementary Fig. 4b and see also Fig. 5a). After treatment with lambda phosphatase (λP) or alkaline phosphatase (AP), both species collapsed into a single band co-migrating with H1 or migrating slightly below H1 (Supplementary Fig. 4b). Thus, H1 likely represented a mixture of non-phosphorylated and low-level phosphorylated Hsf1 species, which were not well resolved on phos-tag gels, while H2 was more highly phosphorylated.

Supplementary References

1. von Plehwe, U. *et al.* The Hsp70 homolog Ssb is essential for glucose sensing via the SNF1 kinase network. *Genes Dev.* **23,** 2102-2115 (2009).
2. Hübscher, V. *et al.* The Hsp70 homolog Ssb and the 14-3-3 protein Bmh1 jointly regulate transcription of glucose repressed genes in Saccharomyces cerevisiae. *Nucleic Acids Res* **44,** 5629-5645 (2016).
3. Hughes Hallett, J. E., Luo, X. & Capaldi, A. P. State Transitions in the TORC1 Signaling Pathway and Information Processing in Saccharomyces cerevisiae. *Genetics* (2014).

Supplementary Figures and Figure Legends

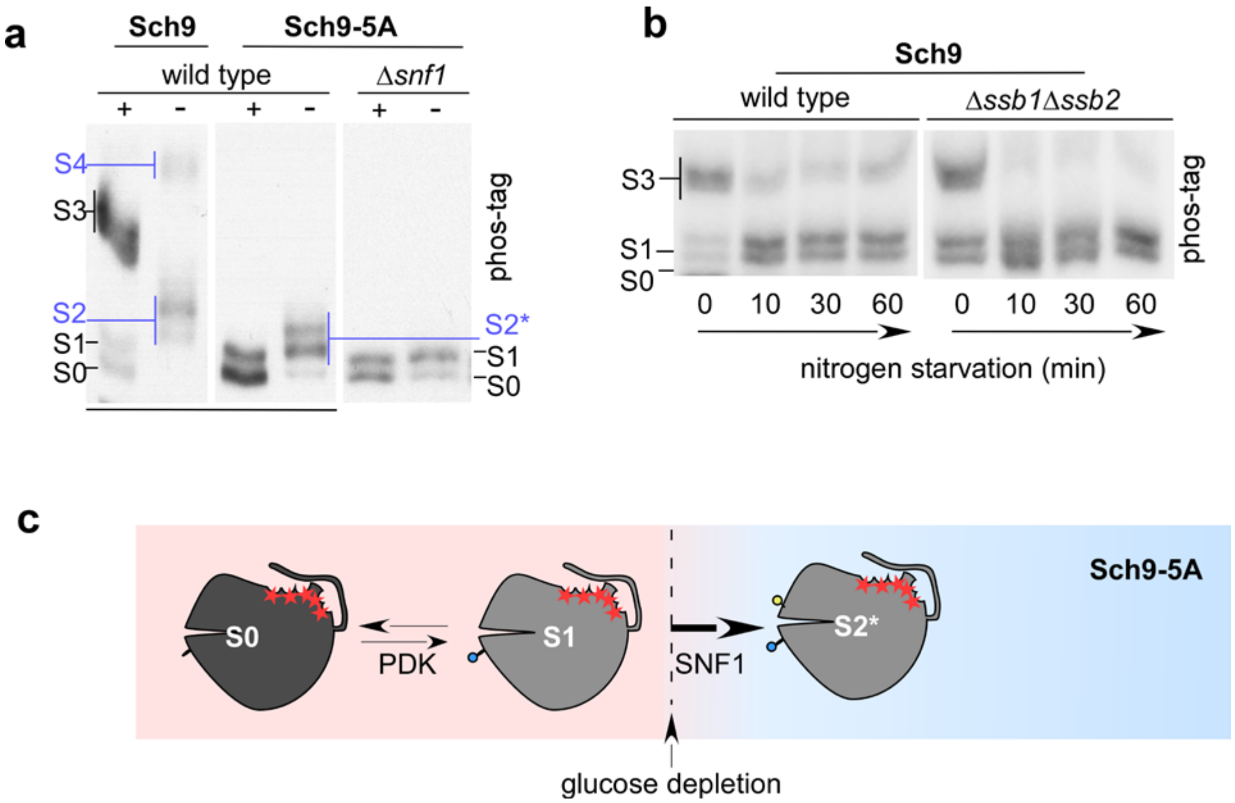

Supplementary Figure 1. Sch9 phosphorylation upon glucose starvation is SNF-dependent and distinct from nitrogen deprivation.

(a,b) Δ*sch9* cells expressing Sch9-FLAG or Sch9-5A-FLAG were grown in glucose-rich conditions (+ glucose) or were depleted for glucose for 10 min (- glucose) (a) or for nitrogen for the indicated times (b). Protein extracts were subsequently analyzed on a phos-tag gel followed by immunoblotting with α-FLAG.

(c) Cartoon summary of Sch9-5A phosphorylation. In glucose-rich conditions Sch9-5A is distributed between S0 and S1, because the residues within the CT, phosphorylated by TORC1 are exchanged to alanines (red stars, see also Fig. 1a and 1f). In glucose-starved conditions the Sch9-5A S0/S1 species are shifted up to Sch9-5A-S2*. This phosphorylation depends on SNF1 and is thus not observed in Δ*snf1* cells. For more details compare Results.

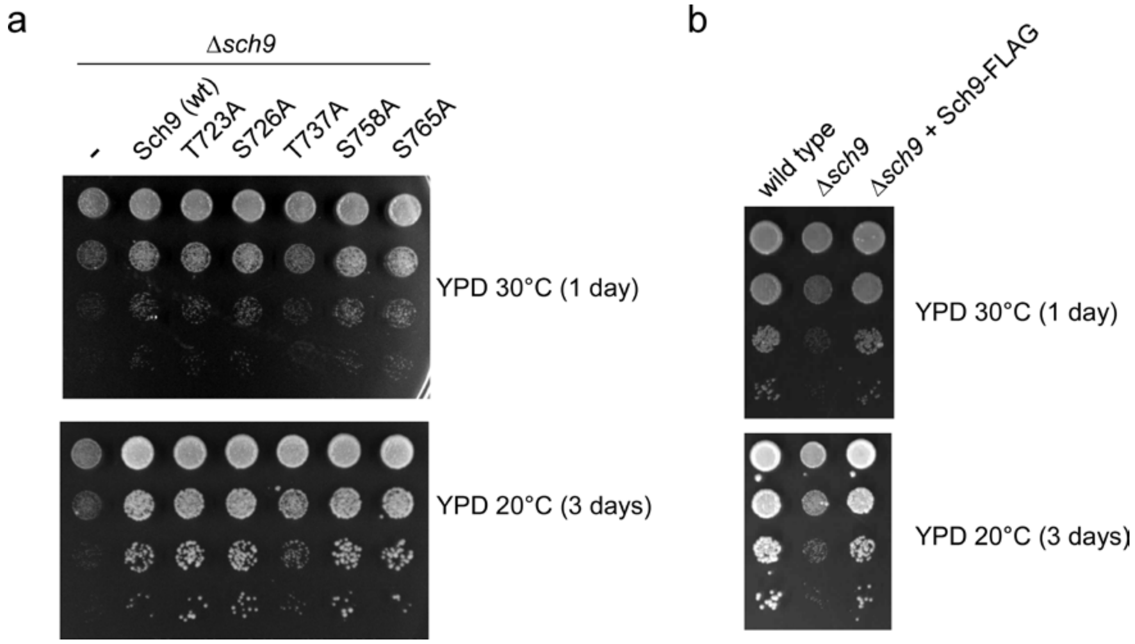

Supplementary Figure 2. Growth analysis of Sch9 C-terminal mutations.

(a) Logarithmically growing cells from Δ*sch9* expressing the indicated versions of Sch9-FLAG from a low copy plasmid were serially diluted, spotted on YPD plates, and were incubated at 30°C or 20°C for the times indicated.

(b) FLAG-tagging Sch9 does not affect growth. Logarithmically growing cells from wild type, Δ*sch9*, or Δ*sch9* expressing Sch9-FLAG from a low copy plasmid were serially diluted, spotted on YPD plates, and were incubated at 20°C or 30°C for the times indicated.

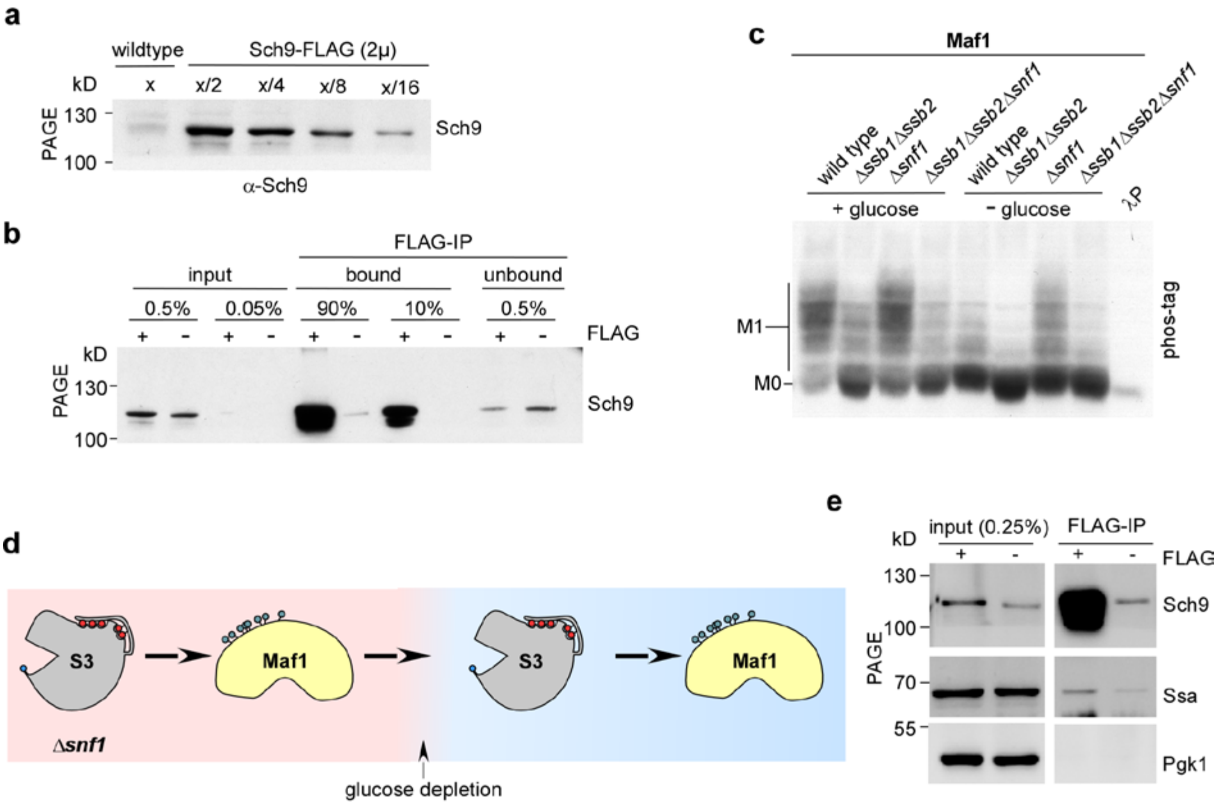

Supplementary Figure 3. FLAG-IP controls and SNF1-dependence of Maf1 phosphorylation.

(a) Relative expression level of endogenous Sch9 and Sch9-FLAG expressed from pYEPlac195-Sch9-FLAG. Glass bead extracts from wild type cells and Δ*sch9* + pYEPlac195-Sch9-FLAG cells were analyzed via immunoblotting with α-Sch9. Dilutions are indicated above the blot.

(b) Estimation of the fraction of Sch9-FLAG bound to FLAG-beads in co-immunoprecipitation experiments. FLAG-IPs were performed with extracts derived from cells expressing Sch9 +/- FLAG as described in Methods. The amount of Sch9-FLAG in the supernatant after immunoprecipitation (unbound) relative to the total Sch9-FLAG (input) was quantified desitometrically using Image Scanner III (GE Healthcare) and AIDA Image analysis software.

(c) Maf1 phosphorylation defect in Δ*ssb1*Δ*ssb2* is not SNF-dependent. The indicated strains were grown in glucose-rich conditions, or were glucose depleted for 10 min. Subsequently protein extracts were analyzed via phos-tag gels followed by immunoblotting with α-Maf1. M0: dephosphorylated Maf1 species, M1: hyper-phosphorylated Maf1 species. λP: lambda phosphatase treated extract.

(d) Cartoon summary of Maf1 phosphorylation in Δ*snf1* cells. Maf1 is phosphorylated by Sch9-S3 in glucose-rich conditions (see Fig. 3). Because in Δ*snf1* cells Sch9-S3 is stable when glucose is depleted (see also Fig. 1e), Maf1 remains hyper-phosphorylated. For more details compare Results.

(e) Ssa co-immunoprecipitates with Sch9. FLAG-IP reactions were performed as described in Fig. 3a, and analyzed using α-Ssa and α-Pgk1. Pgk1, an abundant cytosolic protein, served as a negative control.

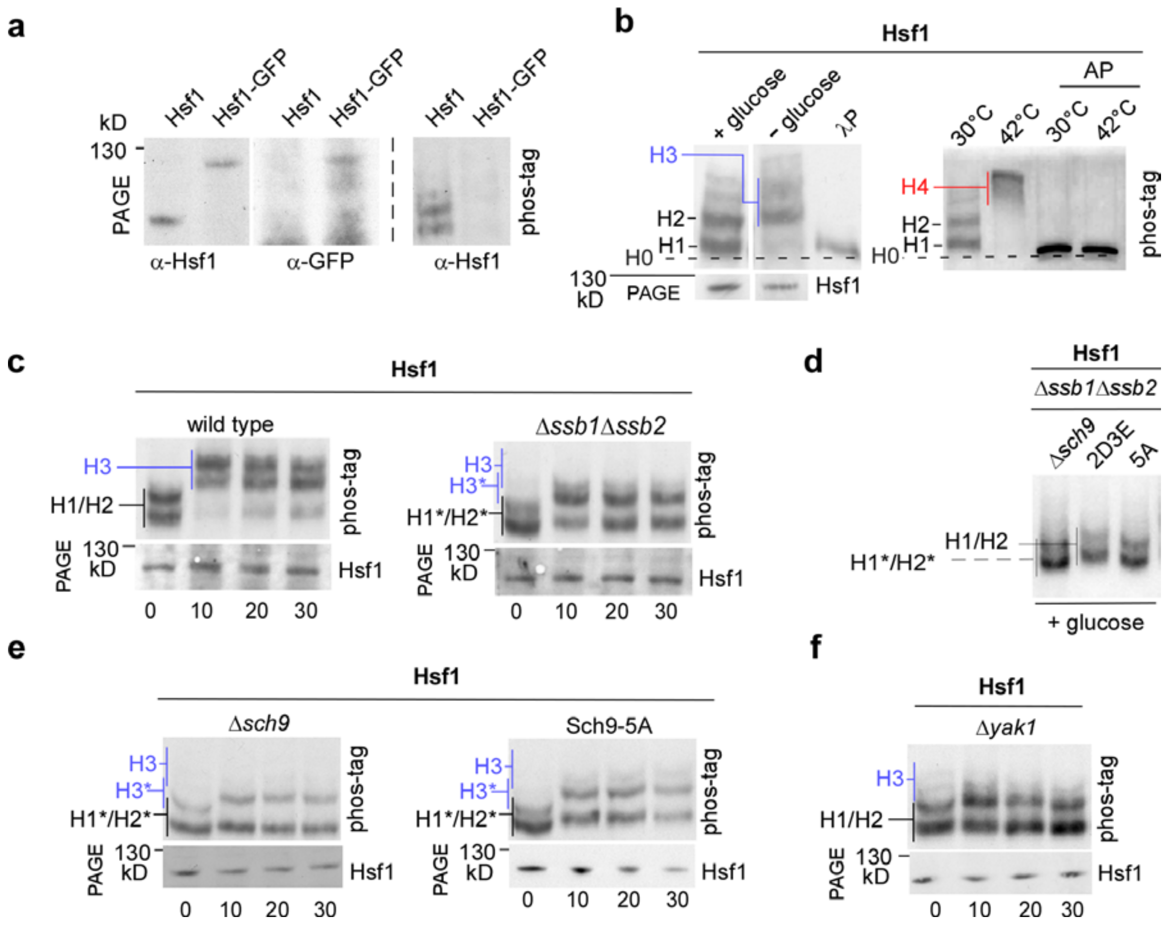

Supplementary Figure 4. Hsf1 phosphorylation in glucose-rich conditions and upon glucose depletion.

(a) α-Hsf1 recognizes specifically Hsf1. Protein extracts from wild type cells or cells expressing Hsf1-GFP were resolved either via phos-tag or Tris-Tricine gels (PAGE), and were subsequently analyzed via immunoblotting with α-Hsf1 and α-GFP.

(b) Hsf1 species resolved by phos-tag gels: H1/H2 (present on glucose), H3 (formed upon 10 min glucose starvation) and H4 (formed upon heat shock at 42°C for 15 minutes) collapse into H0 upon phosphatase treatment. λP: lambda phosphatase treated. AP: alkaline phosphatase treated.

(c,e,f) The phosphorylation pattern of Hsf1 is stable between 5 - 30 min of glucose depletion. The indicated strains were grown in glucose-rich conditions and were then depleted for glucose for 10, 20, or 30 min. Total extracts were subsequently analyzed via phos-tag gels followed by immunoblotting with α-Hsf1. H1/H2: predominant Hsf1 species in glucose-grown wild type; H3: predominant hyper-phosphorylated Hsf1 species upon glucose depletion. H1*/H2*: hypo-phosphorylated Hsf1 species, predominant in glucose-grown Δ*ssb1*Δ*ssb2* cells and cells lacking functional Sch9. H3*: hypo-phosphorylated Sch9 species in glucose-depleted Δ*ssb1*Δ*ssb2* cells and cells lacking functional Sch9. H0: non-phosphorylated Hsf1 species obtained by λ-phosphatase treatment. Please note H0 and H1 are only poorly resolved via phos-tag gel electrophoresis.

(d) Hyperactive Sch9-2D3E rescues H1/H2 formation in Δ*ssb1*Δ*ssb2*. Protein extracts of the indicated strains expressing Sch9-2D3E or Sch9-5A grown in the presence of glucose were analyzed via phos-tag gels using α-Hsf1 as an antibody.

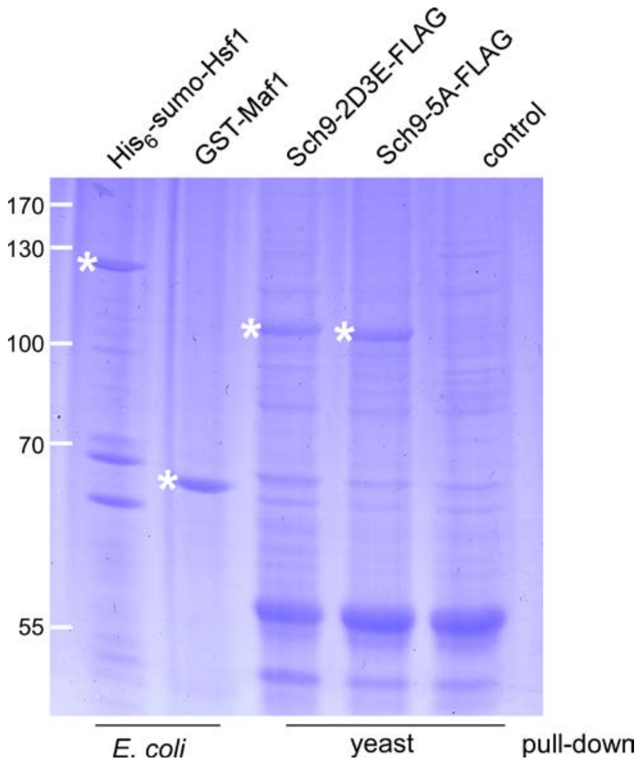

Supplementary Figure 5. Partially purified proteins used in *in vitro* kinase assays. GST-Maf1 and His_6-sumo-Hsf1 were partially purified from *E. coli* as described in Methods. Sch9-2D3E-FLAG, Sch9-5A-FLAG, and as a control untagged Sch9 were enriched via FLAG-IP from yeast extracts as described in Methods. Samples were separated on Tris-Tricine gels and were subsequently stained with Coomassie. Asterisks indicate the enriched proteins of interest.

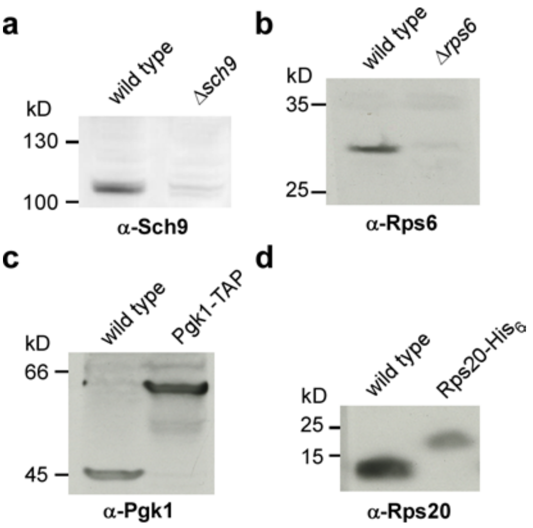

Supplementary Figure 6. Validation of antibodies.
(a,b) α-Sch9 and α-Rps6 antibodies were validated using Δ*sch9* and Δ*rps6* strains respectively. **(c,d)** Since deletion of Pgk1 or Rps20 is lethal, strains expressing the slower migrating TAP-tagged Pgk1 or His_6-tagged Rps20 were used to validate α-Pgk1 and α-Rps20, respectively.

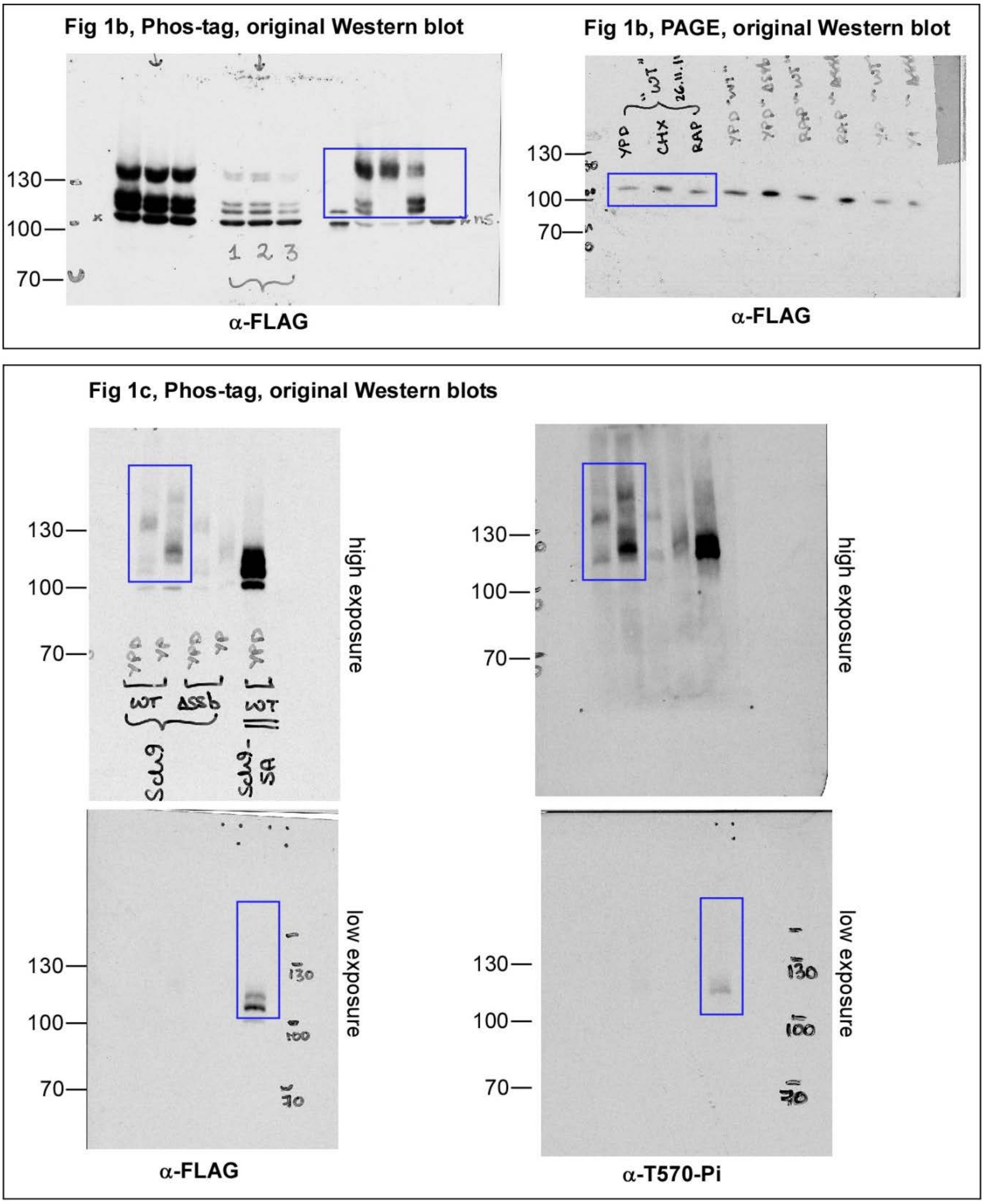

Supplementary Figure 7. Uncropped images of important immunoblots shown in Figure 1. Blue boxes show cropped regions used in Fig. 1.

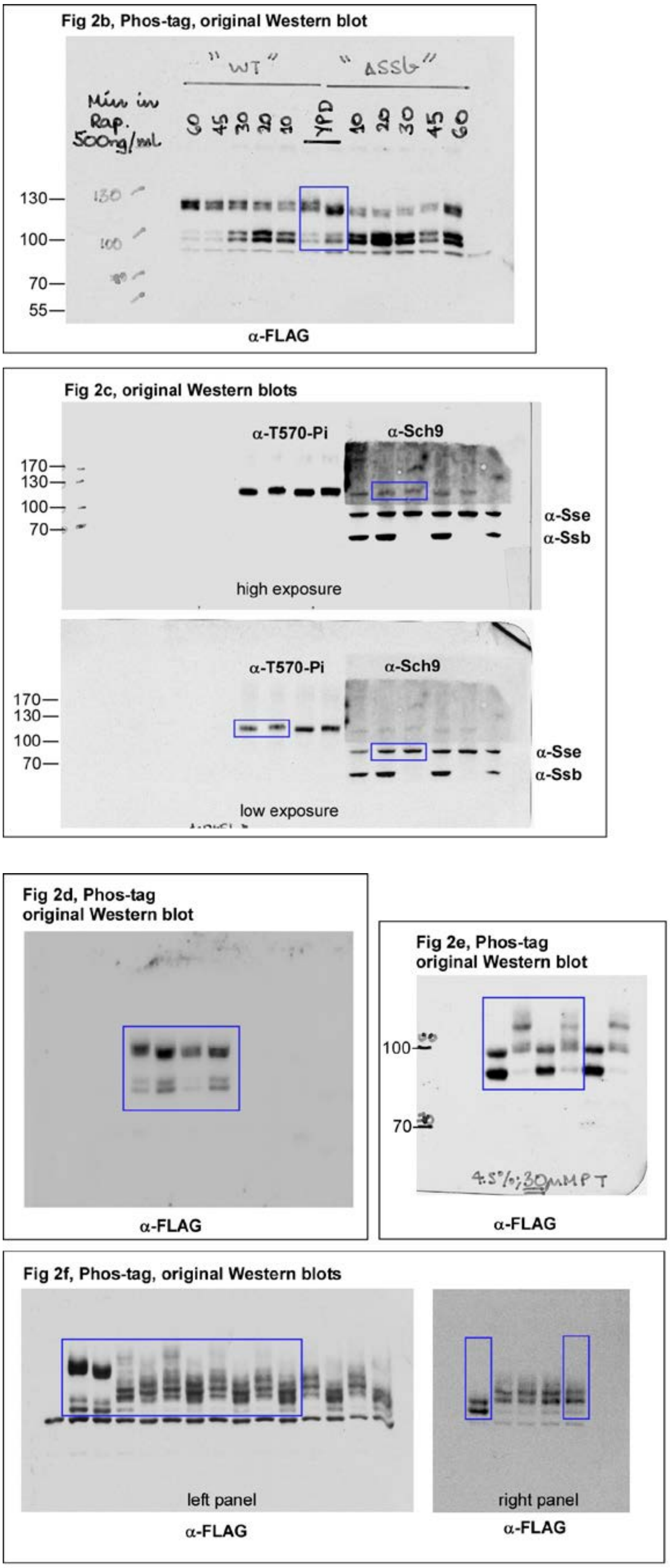

Supplementary Figure 8. Uncropped images of important immunoblots shown in Figure 2. Blue boxes show cropped regions used in Fig. 2.

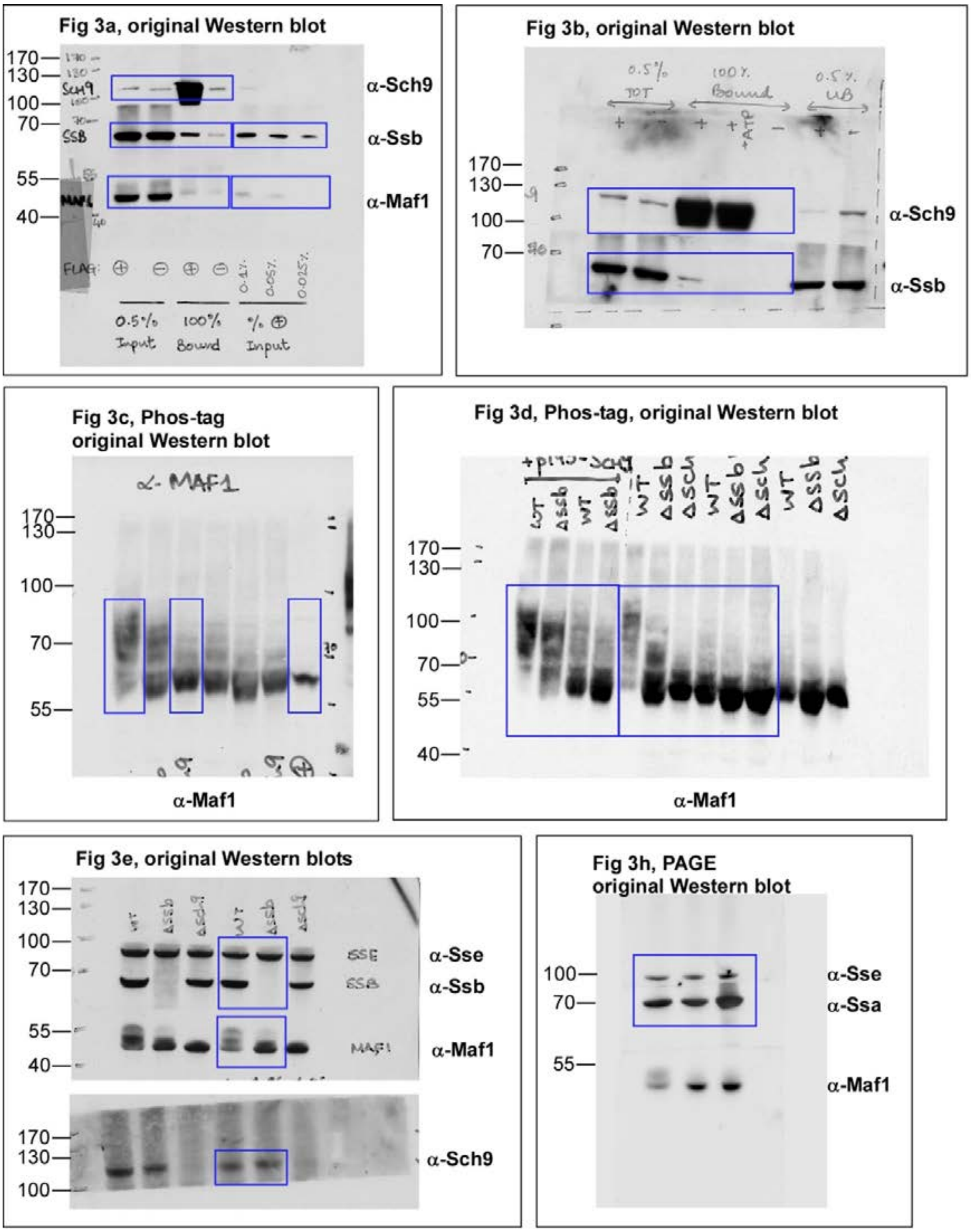

Supplementary Figure 9. Uncropped images of important immunoblots shown in Figure 3. Blue boxes show cropped regions used in Fig. 3.

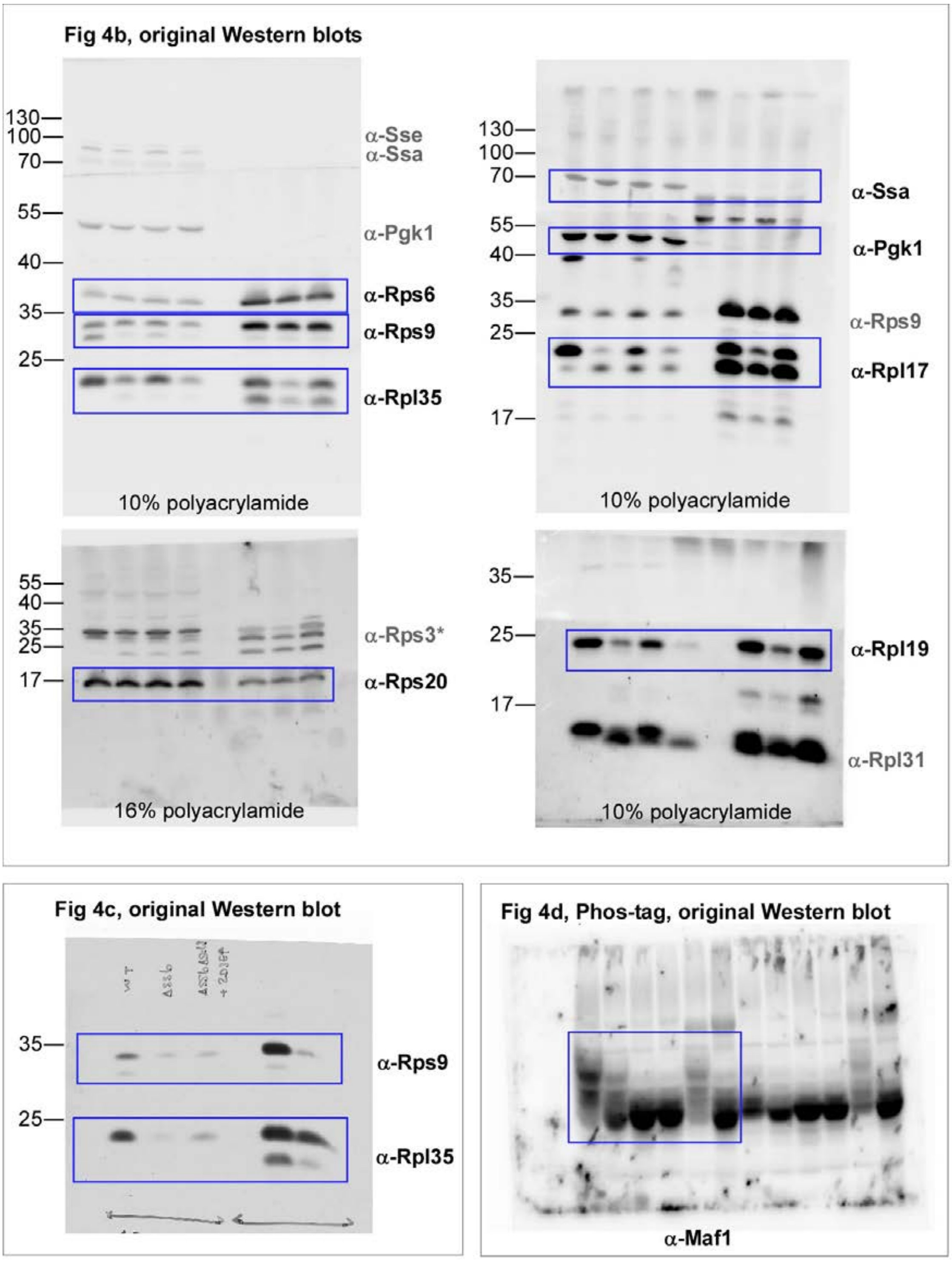

Supplementary Figure 10. Uncropped images of important immunoblots shown in Figure 4. Blue boxes show cropped regions used in Fig. 4. Other antibodies used on different parts of the membrane, which were not included into this study are labeled in grey.

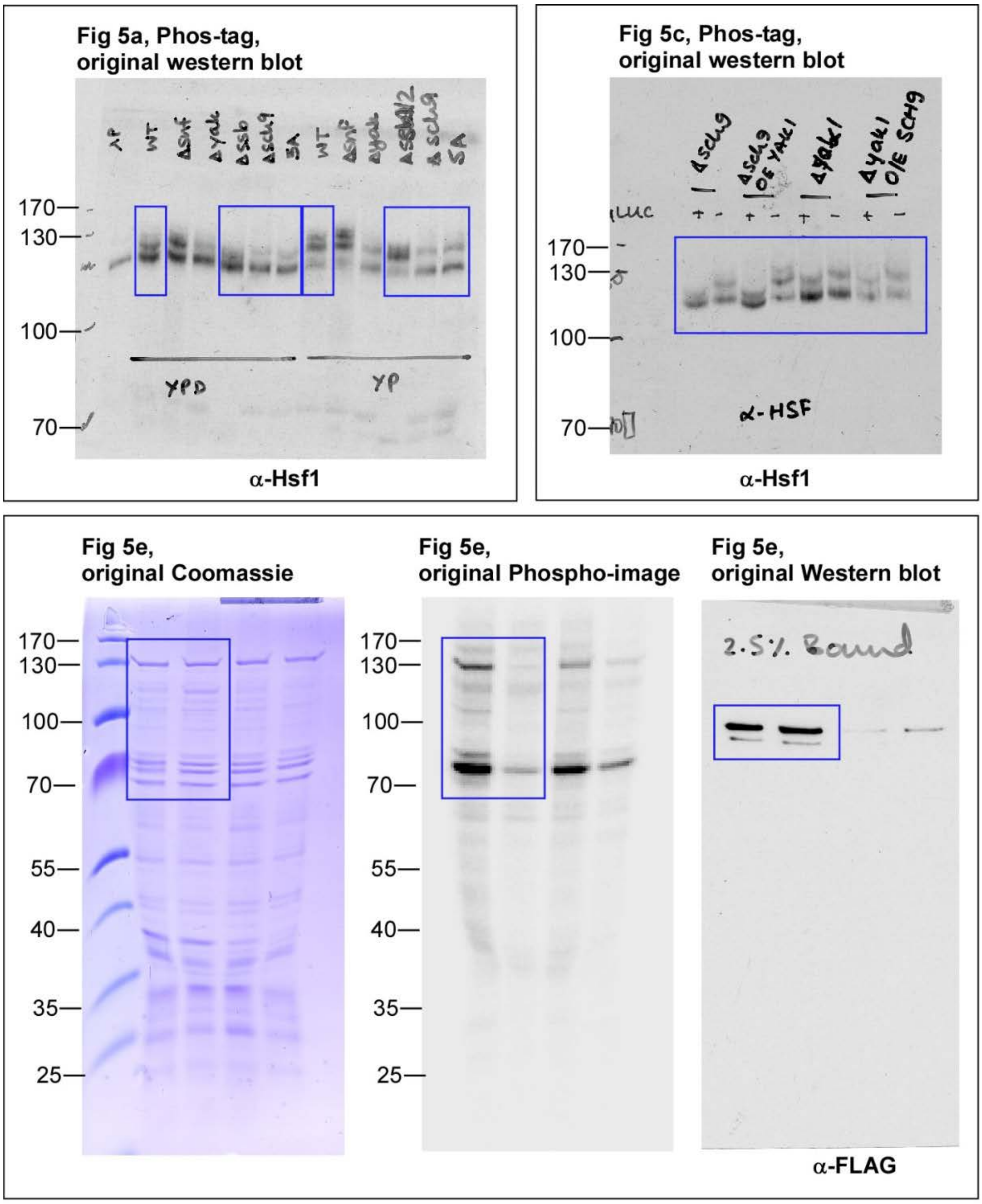

Supplementary Figure 11. Uncropped images of important immunoblots shown in Figure 5. Blue boxes show cropped regions used in Fig. 5.

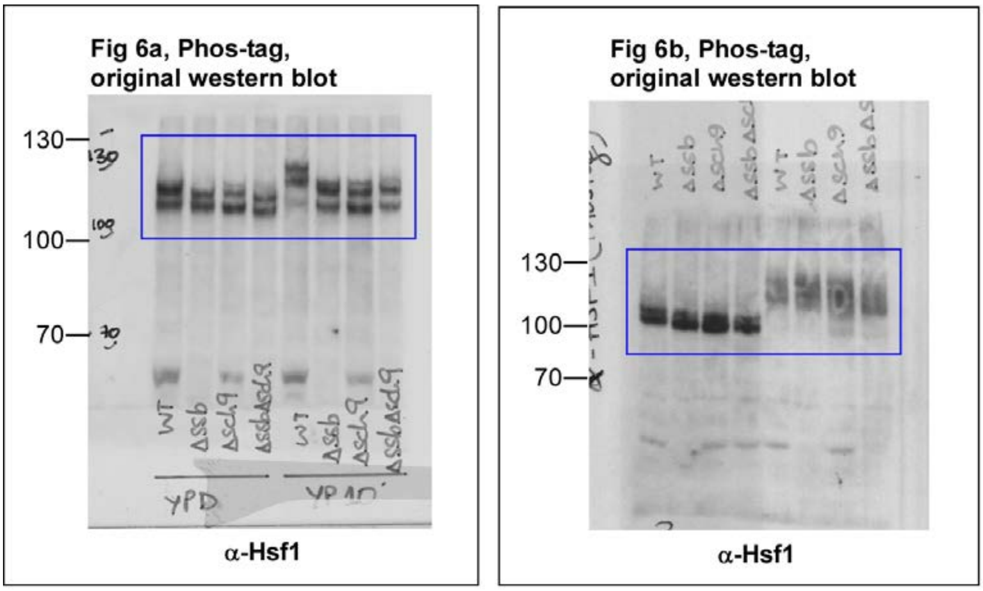

Supplementary Figure 12. Uncropped images of important immunoblots shown in Figure 6. Blue boxes show cropped regions used in Fig. 6.

SUPPLEMENTARY TABLES

Supplementary Table 1. Strains and Plasmids.
S. cerevisiae and *E. coli* strains and expression plasmids employed in the course of the study.

Strains	Genotype	Plasmids	Plasmid Features	Reference
**Yeast Strains**				
MH272-3f a/α (diploid wild type)	*ura3*/*ura3*, *leu2*/*leu2*, *his3*/*his3*, *trp1*/*trp1*, *ade2*/*ade2*	-		von Plehwe et al. 2009
MH272-3f α (haploid wild type)	*ura3 leu2 his3 trp1 ade2*	-		von Plehwe et al. 2009
		pYEPlac195-Sch9	2µ, *URA3*, SCH9+/-300	this study
MH272-3f α Hsf1-GFP	*hsf1:: HSF1-GFP-klTRP1*	-		this study
Δ*ssb1*Δ*ssb2*	*ssb1::ADE2 ssb2::HIS3*	-		von Plehwe et al. 2009
		pYEPlac195-SSA2	2µ, *URA3*, SSA2+/-300	this study
		pYEPlac195-Sch9	2µ, *URA3*, SCH9+/-300	this study
		pYCPlac33-Ssb1-K73A	CEN, *URA3*, SSB1-K73A+/-300	von Plehwe et al. 2009
Δ*sch9*	*sch9::LEU2*	-		this study
		pYCPlac33-Sch9	CEN, *URA3*, SCH9+/-300	this study
		pYCPlac33-Sch9-FLAG	CEN, *URA3*, SCH9-FLAG+/-300	this study
		pYCPlac33-Sch9-5A-FLAG	CEN, *URA3*, SCH9-5A-FLAG+/-300	this study, Urban et al. 2007
		pYCPlac33-Sch9-T570A-FLAG	CEN, *URA3*, SCH9-T570A-FLAG+/-300	this study, Urban et al. 2007
		pYCPlac33-Sch9-K441A	CEN, *URA3*, SCH9-K441A +/-300	this study, Morano and Thiele 1999
		pYCPlac33-Sch9-D556R	CEN, *URA3*, SCH9-D556R+/-300	this study, Morano and Thiele 1999
		pYCPlac33-Sch9-T570A/5A-FLAG	CEN, *URA3*, SCH9-T570A/5A-FLAG+/-300	this study, Urban et al. 2007
		pYCPlac33-Sch9-T723A-FLAG	CEN, *URA3*, Sch9-T723A-FLAG +/-300	this study
		pYCPlac33-Sch9-S726A-FLAG	CEN, *URA3*, Sch9-S726A-FLAG +/-300	this study
		pYCPlac33-Sch9-T737A-FLAG	CEN, *URA3*, Sch9-T737A-FLAG +/-300	this study
		pYCPlac33-Sch9-S758A-FLAG	CEN, *URA3*, Sch9-S758A-FLAG +/-300	this study
		pYCPlac33-Sch9-S765A-FLAG	CEN, *URA3*, Sch9-S765A-FLAG +/-300	this study

		pYEPlac195-Sch9	2µ, *URA3*, SCH9+/-300	this study
		pYEPlac195-Sch9-FLAG	2µ, *URA3*, SCH9-FLAG+/-300	this study
		pYEPlac195-Sch9-2D3E-FLAG	2µ, *URA3*, SCH9-2D3E-FLAG+/-300	this study, Urban et al. 2007
		pYEPlac195-Sch9-5A-FLAG	2µ, *URA3*, SCH9-5A-FLAG+/-300	this study, Urban et al. 2007
		pYEPlac112-FLAG-Yak1	2µ, *TRP1*, FLAG-YAK1+/-300	this study
Δssb1Δssb2Δsch9	*ssb1::ADE2 ssb2::HIS3 sch9::LEU2*	-		this study
		pYCPlac33-Sch9-FLAG	CEN, *URA3*, SCH9-FLAG+/-300	this study
		pYCPlac33-Sch9-5A-FLAG	CEN, *URA3*, SCH9-5A-FLAG+/-300	this study, Urban et al. 2007
		pYCPlac33-Sch9-T737A-FLAG	CEN, *URA3*, Sch9-T737A-FLAG +/-300	this study
		pYEPlac195-Sch9-2D3E	2µ, *URA3*, SCH9-2D3E +/-300	this study, Urban et al. 2007
		pYEPlac195-Sch9-2D3E-FLAG	2µ, *URA3*, SCH9-2D3E-FLAG+/-300	this study, Urban et al. 2007
		pYEPlac195-Sch9-5A	2µ, *URA3*, SCH9-5A +/-300	this study, Urban et al. 2007
Δsnf1	*snf1::kanMX4*	-		von Plehwe et al. 2009
		pYCPlac33-Sch9-T570A/5A-FLAG	CEN, *URA3*, SCH9-T570A/5A-FLAG+/-300	this study, Urban et al. 2007
Δsnf1Δsch9	*snf1::kanMX4 sch9::LEU2*	pYCPlac33-Sch9-FLAG	CEN, *URA3*, SCH9-FLAG+/-300	this study
		pYCPlac33-Sch9-5A-FLAG	CEN, *URA3*, SCH9-5A-FLAG+/-300	this study, Urban et al. 2007
Δssb1Δssb2Δsnf1	*ssb1::ADE2 ssb2::HIS3 snf1::kanMX4*	-		von Plehwe et al. 2009
Δyak1	*yak1::kanMX4*	-		this study
		pYEPlac195-Sch9	2µ, *URA3*, SCH9+/-300	this study
Δzuo1Δssz1	*zuo1::TRP1 ssz1::LEU2*	-	-	Gautschi et al. 2001
Δsse1	*sse1:: HIS3*	-	-	this study
**Bacterial Strains**				
E. coli BL21		pCA528-His_6-sumo-Hsf1	Kan^R ; IPTG inducible	M. Mayer
		pGEX-4T-1-GST-Maf1	Amp^R ; IPTG inducible	Wei et al. 2009

Supplementary Table 2. Primer sequences.
Primers employed for RT-PCR experiments shown in Fig. 6a and 6b.

NAME	SEQUENCE	AMPLICON SIZE (bp)
ACT1-F<br>ACT1-R	*GGCATCATACCTTCTACAAC*<br>*CGATGTTACCGTATAATTCC*	630
BTN2-F<br>BTN2-R	*GGAGGAAAGCAATAAAAG*<br>*CATATGGAATCCGC*	150
CUP1-F<br>CUP1-R	*GTTCAGCGAATTAATTAAC*<br>*GCATTTGTCGTCG*	120
CPR6-F<br>CPR6-R	*GACGATTATCAAGTGC*<br>*CTTTCAAGACGGTGTC*	124
